# Prevalence and associated factors of infertility among 20–49 year old women in Henan Province, China

**DOI:** 10.1186/s12978-021-01298-2

**Published:** 2021-12-20

**Authors:** Shoujing Liang, Yuanhui Chen, Qian Wang, Huanhuan Chen, Chenchen Cui, Xiaohang Xu, Qingwen Zhang, Cuilian Zhang

**Affiliations:** Department of Reproductive Medicine Center, Zhengzhou University People’s Hospital, Henan Provincial People’s Hospital, No. 7 Weiwu Road, Zhengzhou, Henan Province China

**Keywords:** Epidemiology, Infertility, Reproduction, Prevalence, Risk factors

## Abstract

**Background:**

Infertility is a reproductive health problem which affects not only individuals, families and social populations. Recently, the infertility rate in China has a trend of increase year by year, and few studies have reported the infertility rate in Henan Province, China. The aim of this study was to investigate the current prevalence and associated factors of infertility among women of childbearing age in Henan Province, China.

**Methods:**

This cross-sectional study was conducted from March 2019 to October 2019. We sampled 765 women who were 20–49 years old in eight hospitals of four cities in Henan Province, China. This survey included a questionnaire, physical examination, vaginal ultrasound examinations, and serum anti-Mullerian hormone (AMH) assessment, all of which were conducted under uniform standards by trained personnel. According to the data collected from questionnaire, participants were divided into infertile and fertile groups and analyzed associated factors.

**Results:**

Among all the 765 participants in this study, the prevalence of infertility was 24.58%. The prevalence of primary infertility was 6.54%, and the prevalence of secondary infertility was 18.04%. In logistic multivariate regression analyses, infertility was associated with age (p < 0.001), history of gynecological surgery (p < 0.001), sweet food (p = 0.003) and decreased ovarian reserve (DOR) (p < 0.001). After further analyses, factors associated with primary infertility were age of marriage (p = 0.006), age of first sexual intercourse (p = 0.003), long-term air-conditioning environment (p < 0.001), decreased ovarian reserve (p = 0.005) and age (p = 0.002). And factors associated with secondary infertility were history of gynecological surgery (p < 0.001), decreased ovarian reserve (p = 0.002), waist-to-hip ratio (WHR) above 0.85 (p = 0.043), delivery times (p = 0.001) and ages (p < 0.001).

**Conclusion:**

The prevalence of infertility among women aged 20–49 was 24.58% and only 61.17% infertile women sought medical help in Henan Province, China. Age, history of gynecological surgeries and DOR may increase the risk of infertility. Local public health departments and medical professionals need to discharge their duty of reducing the high incidence of infertility and protecting women’s reproductive health.

**Supplementary Information:**

The online version contains supplementary material available at 10.1186/s12978-021-01298-2.

## Plain Language summary

Infertility prevalence rate has increased in the past 30 years. Infertility plagues thousands of women of childbearing age. Although not life-threatening, the detrimental influence of infertility to patients, their families, and society should not be underestimated, especially in China. In order to investigate the prevalence of infertility, determine the associated factors, and promote disease prevention and treatment, we conducted a cross-sectional study among 20–49 year old women in Henan, one of the central provinces of China.

This study distributed 920 questionnaires and collected 803 completed questionnaires. Interviews, questionnaires, and physical and ultrasound examinations were done.

Among all the 765 participants in this study, the prevalence of infertility was 24.58%. The prevalence of primary infertility was 6.54%, and the secondary infertility was 18.04%. Age, history of gynecological surgeries and DOR may increase the risk of infertility.

In conclusion, among women aged 20–49 years in Henan Province, China, the prevalence of infertility in 2019 was 24.58% and 61.17% of infertile women sought medical help.

**Supplementary Information:**

The online version contains supplementary material available at 10.1186/s12978-021-01298-2.

## Background

Infertility is a global reproductive health problem and the prevalence rate increased by 0.37% per year for females and the global disease burden of infertility had increased from 1990 to 2017 [1]. With environment and lifestyle changes, the incidence of infertility might be associated with the delay of marriage and giving birth to the first child [2, 3], environmental pollution [4], and unhealthy lifestyles [5]. Although not life-threatening, the detrimental influence of infertility to patients, their families, and society should not be underestimated. For patients diagnosed with infertility, psychological pressure rises and the relationships between family members deteriorate [6], which may greatly affect the quality of life. In addition, the declining birth rate could potentially worsen the aging problem.

In 2007, international estimated about the prevalence of infertility in developed countries ranged from 3.5 to 16.7%, while in developing countries the prevalence ranged from 6.9 to 9.3% [7]. At present, there were only a few studies about infertility epidemiology in China. According to national survey data from China in 1988, infertility rates were 2.3% in Qinghai Province, 3.7% in Tibet, and 3.7% in Xinjiang, respectively [8]. The prevalence of infertility was found to be 5.1% in Shanghai, China, in a study of 7872 newly married couples in 1992–1993 [9]. In 2010–2011, a population-based study including 25,270 couples found that the prevalence of infertility was 15.5%, and among couples who were actively attempting to become pregnant the rate was 25.0% in China [10]. The prevalence of infertility was about 13.09% in rural northern of China in 2014 [11] and was 11.87% in Guizhou Province in 2012 [12]. Little reported information was available about the prevalence of infertility in Henan Province, a central Chinese province with a large population.

Meanwhile, there was difference in the risk factors of infertility among different studies. A national population-based study in China found that risk factors were irregular menstrual cycle, light menstrual blood volume, history of cervicitis and endometriosis, previous stillbirth and miscarriage and history of operation [10]. A research at a rural site of Northern China showed that risk factors for infertility included body mass index (BMI), state of exercise, amount of menstrual flow, and number of pregnancies and abortions [11]. Another research enrolled 2151 newly married couples in rural region of northern China and found that hepatitis B, epilepsy, diabetes, passive smoke and overweight were associated with infertility [13]. A large-scale community-based study which recruited 12,964 women aged 18–49 years in China reported that women with oligomenorrhea had higher prevalence of infertility [14]. Another study reported that increasing age at menarche was associated with infertility [12].

Assisted reproductive technology (ART) has been shown to effectively help infertile couples to have their own children [15]. In 1994, infertility was listed as the components of reproductive health for the first time at International Conference on Population and Development (ICPD) [16]. In 2004, the World Health Organization’s first global strategy on reproductive health was presented and infertility services became one of the aspects [17]. It is also a reproductive right for infertile couples to have access to ART [18]. One research reported in 2016 by National Health Commission of the People’s Republic of China that there were 451 licensed ART clinics in total in mainland China [19] and another research reported by the Chinese Society of Reproductive Medicine that there were in total 1,211,303 cycles from 133 ART centers in China during 2013–2016 [20]. However, previous study showed that only 55.2% infertile women had a willingness to seek medical help in China [10], which might be attributed to the medical cost and social stigma.

This cross-sectional study investigated the prevalence of infertility and AMH level among women of childbearing age in Henan Province, China, for the first time. It provided detailed information on the willingness of infertile women to seek medical help and associated factors of infertility, all of which could help to know reproductive health of childbearing aged women, provide ART services purposely and promote campaigns on popular science of infertility prevention in Henan Province, China.

## Materials and methods

### Design and study population

This cross-sectional study was carried out from March 2019 to October 2019 in Henan Province, China. The previous survey showed the prevalence of infertility in developing countries was about 9.3% [7]. The formula for simple estimation of sample size was $\frac{{{Z}_{\frac{\alpha }{2}}}^{2}P(1-P)}{{E}^{2}}$ (${Z}_{\frac{\alpha }{2}}=1.64, P=9.3\%$,$E=2\%$). With an allowable error of 2% and a confidence level of 90%, a total sample size of 568 would be required. We assumed 30% non-response rate resulting a required sample size of 811, which was a reference size for us to distribute questionnaire. Multistage stratified random sampling was used. We chose Zhengzhou, Zhumadian, Xinxiang, and Luoyang randomly to represent central, southeastern, northeastern, and western populations, respectively, as they are among the top 10 cities with the largest population in Henan Province, China. Finally, eight hospitals were chosen randomly through these four cities, including two urban hospitals and one rural hospital in Zhengzhou, one rural hospital in Zhumadian, and one urban hospital and one rural hospital in Xinxiang and Luoyang separately. The ratio of the number of participants from rural to urban was approximately 1:1.

Women were recruited through information letters and prints ads in these four cities. Participants could receive free physical and ultrasound examinations, serum AMH test and health counseling. All participants signed an informed consent and each had a unique identification number assigned to protect their privacy. The inclusion criteria for participants were as follows: (1) 20–49 years old; (2) married or cohabitational; (3) local residents. The exclusion criteria for participants were as follows: (1) never had sexual intercourse; (2) had life-threatening major diseases such as hepatic failure, renal failure, malignant tumors, or severe cardiovascular and cerebrovascular diseases; (3) received continuous medical treatment that could affect fertility. This survey was conducted by the Ethics Committee of Henan Provincial People’s Hospital (Zhengzhou, China, NO. SYSZ-LL-2019012410).

### Sample and data collection

**Fig. 1:**
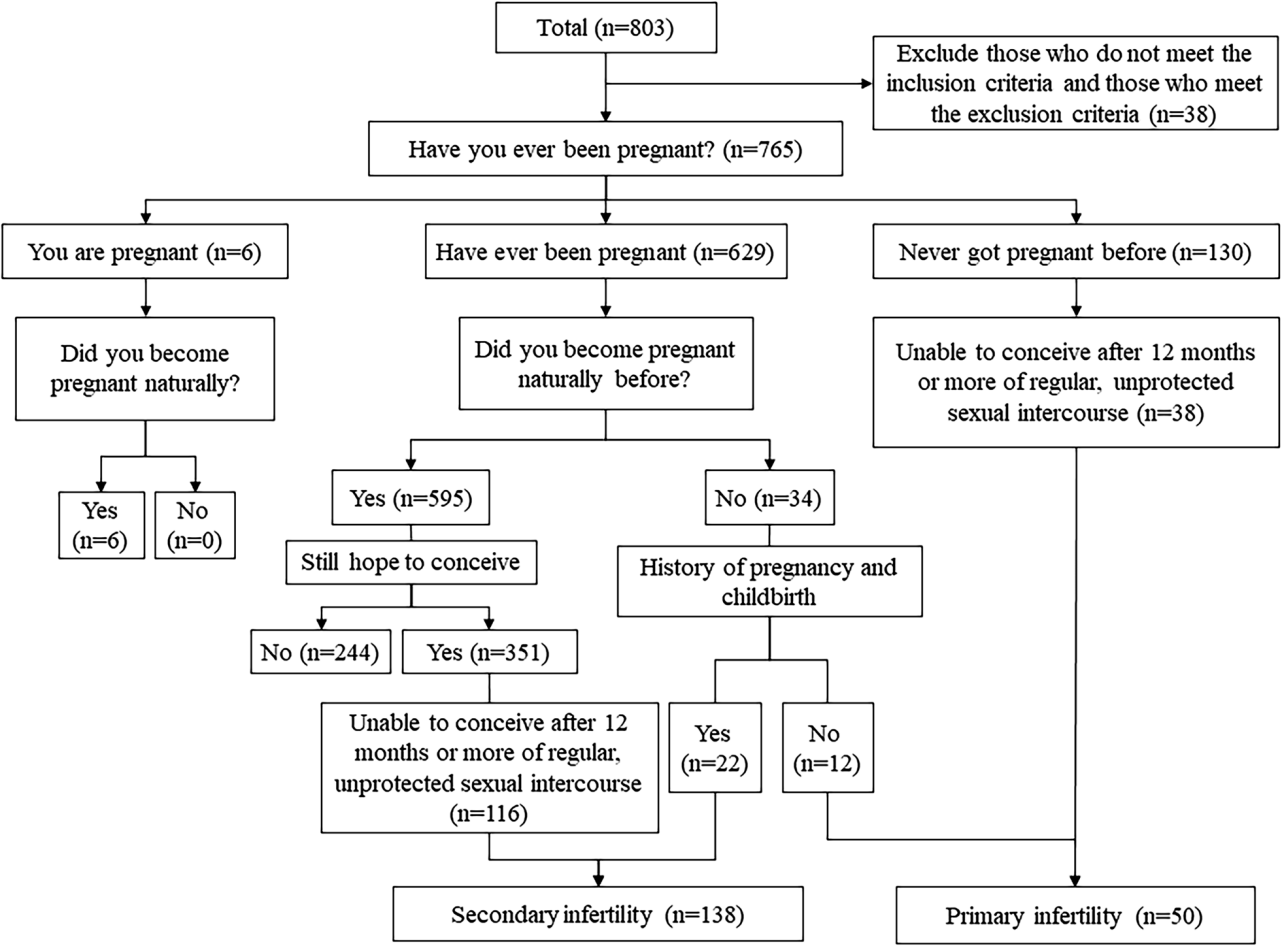
Flow chart of the decision process to identify by questionnaire

We formed a working group under same training to conduce this research, which included three senior oby-gyne specialists and three reproductive medical graduate students. This working group went to these eight hospitals to recruit participants. Publicity by information letters and prints ads were carried out in advance, and all local women who saw these could contact the working group when they arrived. This study includes interviews, questionnaires, physical and ultrasound examinations, and serum sample collections. First, face-to-face interviews were conducted by one specialist. During the interviews, participants would be introduced this research and be informed the pros and cons of participating in this research. If they agree to participate, they should sign an informed consent and continue the next steps. They could opt out of this research at any time but not be count as a valid sample for this study. The next step was to complete the paper questionnaires, which contained a series of questions and divided into three parts, including basic information, marriage and childbirth history, and personal lifestyle habits, under the guidance of this specialist. Meanwhile, participants would have access to free health counseling in this process. Then, physical and ultrasound examinations were performed and recorded by other two specialists. The vaginal ultrasound examinations were done by one, and another recorded the data on the paper questionnaires including sizes of ovaries and uterine, antral follicular counts (AFC), endometrial thickness and special cases such as ovarian cysts and uterine fibroids. Third, serum samples were collected in the morning after fasting for solids and liquids. All serum samples were stored at – 80 °C and tested with uniform standards. This process was done by 3 graduate students of the working group. Supervision and quality control were conducted throughout the entire study. The study adopted double entry mode of paper questionnaire data and were analyzed anonymously. Finally, we distributed 920 paper questionnaires and collected 803 completed paper questionnaires and 800 serum samples. Figure 1 showed the flow chart of the decision process to identify infertile women according to the queries in the questionnaire. After excluding those who did not meet the inclusion criteria and who met the exclusion criteria, the final number of study participants was 765.

Among 765 participants, 6 were pregnant naturally, 629 did become pregnant, and 130 had never gotten pregnant before. According to their history of pregnancy, child birth and contraception, the infertile women were divided into primary infertility and secondary infertility cohorts.

### Definition and standard of diagnosis

Infertility is defined as the failure to achieve a clinical pregnancy after 12 months or more of regular unprotected sexual intercourse according to the World Health Organization [21, 22]. Primary infertility is defined as a woman who has never been diagnosed with a clinical pregnancy and meets the criteria of being classified as being infertile, while secondary infertility is defined as a woman unable to establish a clinical pregnancy who has previously been diagnosed with a clinical pregnancy [23]. The diagnostic criteria applied for polycystic ovary syndrome (PCOS) are the 2003 Rotterdam criteria [24]. The history of gynecological surgery mentioned below includes ovarian cyst removal or oophorectomy, uterine fibroids surgery, uterine mediastinal resection or uterine adhesion lysis, and fallopian tube surgery. Among the frequency mentioned below, ‘often’ means 5–7 times a week and ‘sometimes’ means 3–4 times a week, and ‘seldom’ means 1–2 times a week. ‘Thin’ means that BMI is less than 18.5 kg/m^2^. ‘Normal’ means 18.5 kg/m^2^ ≤ BMI < 24 kg/m^2^. ‘Overweight’ means 24 kg/m^2^ ≤ BMI < 28 kg/m^2^. ‘Obesity’ means that BMI is more than 28 kg/m^2^. The normal reference value of serum AMH level ranges 0.32–8.64 ng/ml for women aged 20–35 years, 0.12–6.08 ng/ml for women aged 36–42 years, and 0.01–3.61 ng/ml for women older than 43 years. Sweet food refers to food with high sugar content including but not limited to fruits such as lychees, grapes and durians, drinks such as cola and bubble tea and snacks such as chocolates, cakes and cookies.

### Statistical analysis

Measurement data used mean ± standard deviation (SD) to describe central tendency and dispersion degree, while count data used frequency and percentage (%) to express. We used Pearson’s Chi-squared test to compare disorder classified data and independent samples T-test to compare the means of two samples. We used binary logistic multivariate regression analysis, and for variable selection we used forward stepwise regression based on maximum likelihood estimation (entry level α = 0.05, elimination level α = 0.1). p < 0.05 indicated that the difference was statistically significant. SPSS version 26.0 was used for statistical processing.

## Results

### Study population

The mean ± SD age was 32.971 ± 0.259 years for the fertile group and 34.957 ± 0.396 years for the infertile group. The difference in mean age between these two groups was statistically significant (p < 0.001). The mean ± SD of BMI was 22.561 ± 0.137 kg/m^2^ for the fertile group and 22.759 ± 0.237 kg/m^2^ for the infertile group. The mean ± SD of WHR was 0.825 ± 0.003 for the fertile group and 0.818 ± 0.005 for the infertile group. The mean ± SD of AMH for the fertile group was 3.734 ± 0.151 ng/ml and 3.557 ± 0.262 ng/ml for the infertile group. The mean ± SD of the age of marriage for the fertile group was 24.359 ± 0.117 years and 25.069 ± 0.235 years for the infertile group.

As shown in Table 1, the difference in the region, age, age of marriage, history of gynecological surgery, the frequency of eating sweet food, long-term stay in air-conditioning environment, ovarian dysfunction, and history of endometriosis between the two groups were statistically significant (p < 0.05). In addition, the prevalence of PCOS was 11.4%.

**Table 1:** Comparison of characteristics of the difference between fertile and infertile group

Characteristic	Fertile		Infertile		p-value
	(n = 577)		(n = 188)		
	n	%	n	%	
Region					
Zhengzhou	124	21.5	62	33.0	0.012
Luoyang	144	25.0	44	23.4	
Xinxiang	161	27.9	46	24.5	
Zhumadian	148	25.6	36	19.1	
Household registry					
Non-agricultural	272	47.1	92	48.9	0.669
Agricultural	305	52.9	96	51.1	
Nationality					
Han nationality	570	98.8	187	99.5	0.425
Minority	7	1.2	1	0.5	
Occupation					
Unemployed	74	12.8	30	16.0	0.541
Head of enterprises and state institutions	79	13.7	29	15.4	
Professional worker	226	39.2	60	31.9	
Clerk	24	4.2	5	2.7	
Service worker	79	13.7	26	13.8	
Agricultural and related worker	13	2.3	7	3.7	
Operator	4	0.7	1	0.5	
Other	78	13.5	30	16.0	
Education					
Middle school and below	75	13	33	17.6	0.391
High school or technical secondary school	91	15.8	25	13.3	
Junior college degree	130	22.5	38	20.2	
Bachelor’s degree and above	281	48.7	92	48.9	
Annual household income (yuan)					
≤ 10,000	139	24.1	42	22.3	0.450
1–20,000	121	21	41	21.8	
2–30,000	101	17.5	32	17.0	
3–50,000	127	22	34	18.1	
≥ 50,000	89	15.4	39	20.7	
Age (years)					
20–24	33	5.7	2	1.1	< 0.001
25–29	158	27.4	22	11.7	
30–34	187	32.4	75	39.9	
35–39	95	16.5	54	28.7	
40–44	71	12.3	24	12.8	
45–49	33	5.7	11	5.9	
BMI (kg/m^2^)					
Thin	39	6.8	16	8.5	0.486
Normal	373	64.6	110	58.5	
Overweight	125	21.7	48	25.5	
Obese	40	6.9	14	7.4	
WHR					
≤ 0.85	348	60.3	126	67.0	0.100
> 0.85	229	39.7	62	33.0	
Irregular menstruation					
No	403	69.8	122	64.9	0.204
Yes	174	30.2	66	35.1	
Dysmenorrhea					
No	330	57.2	103	54.8	0.563
Yes	247	42.8	85	45.2	
Age of marriage (years)					
≤ 24	303	52.5	78	41.5	0.041
25–29	255	44.2	98	52.1	
≥ 30	19	3.3	12	6.4	
Age of first sexual intercourse (years)					
< 20	78	13.5	18	9.6	0.197
20–25	382	66.2	123	65.4	
> 25	117	20.3	47	25.0	
PCOS					
No	511	88.6	167	88.8	0.920
Yes	66	11.4	21	11.2	
History of gynecological surgery					
No	538	93.2	149	79.3	< 0.001
Yes	39	6.8	39	20.7	
Smoking					
No	430	74.5	147	78.2	0.592
No, secondhand smoke	139	24.1	39	20.7	
Yes	8	1.4	2	1.1	
Drinking					
No	503	87.2	173	92.0	0.072
Yes	74	12.8	15	8.0	
Sweet food					
Often	124	21.5	34	18.1	0.006
Sometimes	321	55.6	89	47.3	
Seldom	132	22.9	65	34.6	
Fried food					
Often	91	15.8	20	10.6	0.196
Sometimes	368	63.8	124	66.0	
Seldom	118	20.5	44	23.4	
Work pressure					
A little	107	18.5	41	21.8	0.482
Some	215	37.3	68	36.2	
Medium	237	41.1	70	37.2	
A lot	18	3.1	9	4.8	
Long-term air-conditioning environment					
No	203	35.2	86	45.7	0.009
Yes	374	64.8	102	54.3	
Renovation within six months					
No	469	81.3	153	81.4	0.976
Yes	108	18.7	35	18.6	
DOR					
No	561	97.2	164	87.2	< 0.001
Yes	16	2.8	24	12.8	
History of colpitis					
No	404	70.0	139	73.9	0.304
Yes	173	30.0	49	26.1	
History of cervicitis					
No	488	84.6	159	84.6	1.000
Yes	89	15.4	29	15.4	
History of pelvic inflammation					
No	470	81.5	154	81.9	0.888
Yes	107	18.5	34	18.1	
History of uterine fibroids					
No	564	97.7	179	95.2	0.071
Yes	13	2.3	9	4.8	
History of endometriosis					
No	565	97.9	179	95.2	0.048
Yes	12	2.1	9	4.8	
History of artificial abortion					
No	351	60.8	122	64.9	0.319
Yes	226	39.2	66	35.1	
History of medical abortion					
No	469	81.3	161	85.6	0.174
Yes	108	18.7	27	14.4	
History of spontaneous abortion					
No	526	91.2	164	87.2	0.116
Yes	51	8.8	24	12.8	
History of ectopic gestation					
No	557	96.5	176	93.6	0.083
Yes	20	3.5	12	6.4	

p-value was based on Person’s Chi-squared test

Additional file 1: Table S1 showed that serum AMH level decreased with the increase of age both in fertile group and infertile group, while there were no significant differences between these two groups in different age ranges.

Additional file 1: Fig. S1A showed that the infertility rate rose initially and then decreased for every 5 years added to women’s age. Women aged 35–39 years had the highest infertility rate. When the participants were grouped by age of marriage, the infertility rate increased as the age of marriage increases. Additional file 1: Fig. S2B showed that among all participants the contraceptive prevalence rate was high at all ages, and the rate of women who still hope to conceive decreases as they get older.

### Prevalence of infertility

The prevalence of infertility in this study cohort was 24.58%. The prevalence of primary infertility and secondary infertility was about 6.54% and 18.04%, respectively. Among the infertile women (Table 2), the difference between primaryand secondary infertility was statistically significant by household registry, age, age of marriage, age of first sexual intercourse, and history of cervicitis (p < 0.05). Among those women who had primary infertility, more were from agricultural households than non-agricultural households, while for secondary infertility women the situation was reversed. And more secondary infertility women had a history of cervicitis.

**Table 2:** Comparison of characteristics of primary and secondary infertile groups

Characteristic	Primary infertility (n = 50)		Secondary infertility (n = 138)		p-value
	n	%	n	%	
Household registry					
Non-agricultural	17	34.0	75	54.3	0.014
Agricultural	33	66.0	63	45.7	
Age (years)					
20–24	2	4.0	0	0.0	< 0.001
25–29	16	32.0	6	4.4	
30–34	21	42.0	54	39.1	
35–39	9	18.0	45	32.6	
40–44	2	4.0	22	15.9	
45–49	0	0.0	11	8.0	
Age of marriage (years)					
≤ 24	15	30.0	63	45.7	0.014
25–29	28	56.0	66	50.7	
≥ 30	7	14.0	5	3.6	
Age of first sexual intercourse (years)					
< 20	4	8.0	14	10.1	< 0.001
20–25	23	46.0	100	72.5	
> 25	23	46.0	24	17.4	
History of cervicitis					
Yes	3	6.0	26	18.8	0.031
No	47	94.0	112	81.2	

p-value was based on Person’s Chi-squared test

Additional file 1: Fig. S2 showed that among infertile women 61.17% went to hospital seeking medical help regardless of the outcome after treatment. From data obtained, the main causes of infertility were fallopian tube factor (25.22%), ovulation disorders (28.70%), endometriosis (6.09%), male factor (13.91%), and unexplained factors (23.48%).

### Factors associated with infertility

Table 3 showed the odds ratio (OR) and 95% confidence interval.

**Table 3:** Logistic multivariate regression analyses of factors associated with infertility

Characteristic	p-value	Unadjusted OR (95% CI)	p-value	Adjusted OR (95% CI)
Age (years)				
	< 0.001		< 0.001	
20–24		Ref		Ref
25–29	0.276	2.297(0.515–10.249)	0.587	1.523 (0.334–6.942)
30–34	0.011	6.618(1.549–28.274)	0.031	5.038 (1.163–21.830)
35–39	0.003	9.379 (2.165–40.622)	0.011	6.862 (1.557–30.248)
40–44	0.025	5.577 (1.244–25.007)	0.109	3.49 (0.758–16.070)
45–49	0.035	5.500 (1.131–26.756)	0.174	3.096 (0.607–15.798)
History of gynecological surgery				
No		Ref		Ref
Yes	< 0.001	3.611 (2.235–5.832)	< 0.001	3.063 (1.819–5.159)
Sweet food				
	0.006		0.003	
Seldom		Ref		Ref
Sometimes	0.003	0.563 (0.386–0.822)	0.002	0.517 (0.342–0.781)
Often	0.017	0.557 (0.344–0.902)	0.008	0.494 (0.294–0.830)
DOR				
No		Ref		Ref
Yes	< 0.001	5.131 (2.662–9.889)	< 0.001	3.835 (1.908–7.711)
Constant			0.003	0.109

(CI) for the factors associated with infertility in logistic multivariate regression analyses. Unsurprisingly, infertility was associated with age as shown in logistic regression analysis. Compared with women aged 20–24 years, the adjusted OR for women aged 30–34 years was 5.038 (95% CI, 1.163–21.83) and the adjusted OR for women aged 35–39 years was 6.862 (95% CI, 1.557–30.248). Gynecological operative history was associated with infertility (Adjusted OR, 3.063, 95% CI, 2.145–5.930). Compared with women who seldom eat sweet food, the adjusted OR for women who eat sweet food sometimes was 0.517 (95% CI 1.238–23.207), and who eat sweet food often was 0.494 (95% CI 0.294–0.83). In addition, ovarian dysfunction was associated with infertility (Adjusted OR, 3.835, 95% CI 1.908–7.711).

Further, there were differences among factors associated with infertility, primary infertility and secondary infertility. In Table 4, factors associated with primary infertility were age of marriage (p = 0.006), age of first sexual intercourse (p = 0.003), long-term air-conditioning environment (p < 0.001), decreased ovarian reserve (p = 0.005) and age (p = 0.002). In single-factor analysis, household register (p = 0.050), history of gynecological surgery (p = 0.006) and sweet food (p = 0.022) might have association to primary infertility but all of them were excluded when binary logistic multivariate regression analyses were carried out. In Table 5, factors associated with secondary infertility were history of gynecological surgery (p < 0.001), decreased ovarian reserve (p = 0.002), WHR above 0.85 (p = 0.043), delivery times (p = 0.001) and ages (p < 0.001). In single-factor analyses, history of ectopic gestation (p = 0.005) might be associated with secondary infertility but it was excluded when binary logistic multivariate regression analyses were carried.

**Table 4:** Logistic multivariate regression analyses of factors associated with primary infertility

Characteristic	p-value	Unadjusted OR (95% CI)	p-value	Adjusted OR (95% CI)
Age of marriage (year)				
	< 0.001		0.006	
≤ 24		Ref		Ref
25–29	0.024	2.102 (1.103–4.005)	0.373	1.434 (0.649–3.168)
≥ 30	< 0.001	7.117 (2.651–19.107)	0.002	6.258 (1.962–19.956)
Age of first sexual intercourse (years)				
	< 0.001		0.003	
< 20		Ref		Ref
20–25	0.867	1.098 (0.371–3.248)	0.648	1.302 (0.42–4.035)
> 25	0.018	3.752 (1.257–11.201)	0.017	4.696 (1.326–16.635)
Long-term air-conditioning environment				
No		Ref		Ref
Yes	0.007	0.452 (0.253–0.806)	< 0.001	0.289 (0.152–0.547)
DOR				
No		Ref		Ref
Yes	0.006	3.364 (1.407–8.045)	0.005	3.987 (1.52–10.456)
Age (year)				
	0.030		0.002	
20–29		Ref		Ref
30–39	0.268	0.713 (0.392–1.298)	0.007	0.395 (0.201–0.779)
40–49	0.009	0.142 (0.003–0.619)	0.003	0.103 (0.023–0.462)
Constant			< 0.001	0.095

**Table 5:** Logistic multivariate regression analyses of factors associated with secondary infertility

Characteristic	p-value	Unadjusted OR (95% CI)	p-value	Adjusted OR (95%CI)
History of gynecological surgery				
No		Ref		Ref
Yes	< 0.001	2.937 (1.772–4.870)	< 0.001	2.695 (1.548–4.695)
DOR				
No		Ref		Ref
Yes	< 0.001	3.690 (1.913–7.114)	0.002	3.145 (1.532–6.455)
WHR				
≤ 0.85		Ref		Ref
> 0.85	0.027	0.637(0.428–0.950)	0.043	0.650 (0.428–0.987)
Delivery times				
	< 0.001		0.001	
0		Ref		Ref
1	< 0.001	3.987 (2.250–7.064)	< 0.001	3.247 (1.753–6.015)
2	0.019	2.112 (1.134–3.936)	0.097	1.772 (0.902–3.484)
3	0.003	4.182 (1.606–10.888)	0.025	3.220 (1.160–8.939)
Age (year)				
	< 0.001		< 0.001	
20–29		Ref		Ref
30–39	< 0.001	4.175(2.357–7.394)	< 0.001	3.359 (1.827–6.174)
40–49	0.002	2.924(1.481–5.773)	0.036	2.170 (1.052–4.479)
Constant			< 0.001	0.039

## Discussion

Reports about the prevalence of infertility are rare currently in Henan Province, a populous province in central China. This study is the first to investigate prevalence of infertility among 20–49 year old women in Henan Province, China. Infertility rates vary from country to country, and change all the times. In the United States, the infertility prevalence rate of women aged 15–44 years in 2002 was 15.5% (95% CI, 8.6–27.5%) [25]. In Canada, the infertility rate ranged from 11.5% (95% CI, 10.2–12.9) to 15.7% (95% CI, 14.2–17.4) in 2009 [26]. In Britain, during 2010–2012 the prevalence of infertility in women aged 16–74 years was 12.5% (95% CI, 11.7–13.3) [27]. While in Turkey, the infertility rate decreased from 12.0% to 8.6% in the 1993–2003 period [28]. The prevalence of infertility was 17.3% in an urban area of Iran [29] and 14.2% in tribal communities of Central India [30]. A cross-sectional study showed that estimated percentage of infertility was 31.1% in Nigeria [31].The infertility rates in China were increasing year by year in general [8–14]. China is a developing country with a vast territory and a large population. The infertility rates will be different in different provinces of China. In this study, we conducted a preliminary analysis based on the general population in Henan Province, China. A self-reported questionnaire was used, which was been found to be a useful measure for quantifying fertility problems experienced in the community [32]. Questionnaire can reduce survey costs and increase survey efficiency, but it can also cause recall bias. We designed a series of questions to determine infertility and specifically to minimize recall bias.

The crude prevalence of infertility in this study cohort was 24.58%, which was close to the infertility rate of 25.0% among women attempting to become pregnant, but had increased when compared to the prevalence of infertility among women overall during 2010–2011 in China [10]. The prevalence of secondary infertility (18.04%) was higher than the prevalence of primary infertility (6.54%). This might be due to the fact that older generation in Henan Province got married and gave birth to their first child early because of the traditional family view. We also found that the prevalence of PCOS (11.4%) was higher than the rate (5.6%) of a Chinese national study in 2013 [33] but was similar to the rate (11.2%) of Chengdu Province, China [34]. PCOS is a common endocrine disorder of women and about two-thirds of them will not ovulate regularly [35]. But there was no difference of PCOS rate between fertile and infertile group. Further studies need to focus on population with PCOS if we want to know more about the association between infertility and PCOS. AMH is one of markers for ovarian reserve [36]. AMH, produced by granulosa cells of small ovarian follicles, decreases with increasing age [37], and our results were observed as expected. However, a global reference range standard for AMH has not been established yet. The reference ranges (5–95th percentiles) for AMH in Chinese women based on a large population study were 2.06–12.66, 1.77–13.83, 1.48–11.45, 0.87–9.76, 0.56–9.49, and 0.08–5.70 ng/ml for the age 20–24, 25–29, 30–32, 33–36, 37–39, and 40–54 groups, respectively [38].

The occurrence of infertility is related to various factors. In this study, multivariable logistic regression analyses showed that age, history of gynecological surgery, eating sweet food, and ovarian dysfunction were associated with infertility. Further analyses demonstrated that age of marriage, age of first sexual intercourse, long-term air-conditioning, decreased ovarian reserve and age were associated with primary infertility and history of gynecological surgery, decreased ovarian reserve, WHR above 0.85, delivery times and age were associated with secondary infertility. It is universally acknowledged that fertility declines as age grows, and research reported that fertility starts declining approximately at age 32 years and rapidly declines after age 37 years [39]. In this study, age has correlation with infertility obviously. One possible reason why the prevalence of infertility did not increase with age was that the desire of pregnancy decreased and the rate of protected sexual intercourse was high in women aged 40–49 years, so some potential infertile women aged 40–49 years were ignored according to our decision process. However, it seems that correlations between age with primary infertility and age with secondary infertility were different. It could be explained that young infertile women were more likely to develop primary infertility than secondary infertility possibly. Further, age of marriage and age of first sexual intercourse might have a more important effect than age on primary infertility. After adjusted effects of age, the trends could be observed that women getting married after age 30 years and having first sexual intercourse after age 25 years might have a higher risk of primary infertility. While women aged 30–39 years and 40–49 years were 3.359 (1.827–6.174) and 2.170 (1.052–4.479) times, respectively, more likely to develop secondary infertility than women aged 20–29 years.

The diagnosis of DOR was based on information from questionnaires filled out by participants of this study. Though recall bias did exist, DOR was still one of risk factors of infertility, primary infertility and secondary infertility according to our results. DOR means that response of child-bearing women to ovarian stimulation or fecundity is reduced when compared with women of same age [40]. Environmental factors, smoking, chemotherapy and radiation may be risk factors of DOR [2, 41], which are factors that childbearing age women need to pay attention to.

History of gynecological surgery was another associated factor for infertility, especially for secondary infertility. One research reported that AMH levels decreased after surgery on ovaries, which indicated that surgery might reduce ovarian reserve [42]. Another research reported that myomectomy, as well as the coexistence of pelvic infection and pelvic adhesiolysis reduced the chance of conception among women aged above 30 years [43]. It was interesting that fertile women seemed to prefer eating sweet food more than infertile women, but it was excluded when further analyses were done. Further researches are needed in order to find whether eating different kinds of sweet food have relevance to infertility. Long-term air-conditioning environment might become one of protective factors of primary infertility in our study, whereas the specific correlations need further explorations. Besides, WHR above 0.85 always refers to central obesity among Chinese women, which might become one of protective factors of secondary infertility in our study and was seemingly opposite of the view that abdominal obesity may impair fertility through associated metabolic disorders [44]. Other study demonstrated that central adiposity was not associated with fertility [45]. One possible explanation was that WHR was just one of parameters for evaluating central obesity or central adiposity and most of participants had the normal BMI in this study. Maybe more parameters such as waist circumference (WC), waist-to height ratio (WHtR) and A body Shape Index (ABSI) need to be considered to explore the relationship between central obesity and infertility. Chinese women with high BMI are uncommon when compared to women in western countries. In that case, there was no significant difference between infertile and fertile groups in BMI. Nonetheless, it could not deny the adverse effect of obesity on fertility [46, 47]. For obese infertile women, exercise and weight loss are necessary in order to improve fertility and outcomes of ART [44–46, 48]. In addition, whether delivery times have relevance to secondary infertility needs further discussions. The one-child policy was not strictly enforced in Henan Province, China. Age, abortion, the way of delivery, and other relevant factors need to be considered when further studies are carried out.

It has been demonstrated that smoke exposure and tobacco have a serious impact on mammal reproductive health [49, 50]. It is possible that the number of women who smoked was too small to show a relevance.

Among participants, 61.17% infertile women went to hospital seeking medical help, which was a little higher than 56% according to 25 population surveys sampling 172,413 women [7]. This suggests that local public health departments and medical professionals need to strengthen the promotion of relevant knowledge to help women of childbearing age in Henan Province, China, to protect their fertility and seek medical help in time when suspected infertility occurs, as well as to enhance their confidence in ART to treat infertility. Ovulation disorders (28.70%) and fallopian tube factor (25.22%) were two main causes of infertility, which are factors need to pay more attention to for women in Henan Province, China. In short, we advise women of childbearing age to get married and have children at an appropriate early age. The risk of primary infertility may increase if women getting married at age above 30 years. A study highlighted the consequence of postponing parenthood for those who experienced infertility in their mid to late thirties [3]. Under the China’s three-child policy, the effect of age on secondary infertility will be more prominent. Meanwhile, gynecological surgeries need to be considered carefully, especially surgeries on ovaries and fallopian tubes. Further, stay away from possible factors contribute to DOR such as smoking and radiation and live a healthy life.

## Conclusion

In conclusion, among women aged 20–49 years in Henan Province, China, the prevalence of infertility in 2019 was 24.58%, and the prevalence of secondary infertility (18.04%) was higher than primary infertility (6.54%). The rate of infertile women seeking medical help (61.17%) still had possibility to increase in future by strengthening the promotion of relevant fertility knowledge and healthcare awareness. Age, history of gynecological surgeries and DOR may increase the risk of infertility, all of which are factors needed to pay attention to. Encouraging age-appropriate marriage and childbearing, calling for timely medical examination for suspected infertility, and advocating a healthy lifestyle are things that local public health departments and reproductive professions need to do next.

## Supplementary Information

**Additional file 1.** **Supplement Table S1.** Mean and standard deviation of serum AMH grouped by age. **Supplement Figure S1.** (A) The changes of infertility rate with age and age of marriage. The infertility rate = The number infertile / The total number by age group (no participants got married at 35-39 years or 45-49 years old). (B) The different rate of women who had protected sexual intercourse and who still hoped to conceive in the future by age group. **Supplement Figure S2.** (A) The percentage of infertile women went to hospital seeking medical help. (B) The main cause of infertility according to the women who sought medical help.

## Data Availability

All data generated or analyzed during this study are included in this published article.

## Abbreviations

AMH: Anti-Mullerian hormone
DOR: Decreased ovarian reserve
WHR: Waist-to-hip ratio
BMI: Body mass index
ART: Assisted reproductive technology
AFC: Antral follicular counts
PCOS: Polycystic ovary syndrome
SD: Standard deviation
OR: Odds ratio
CI: Confidence interval
WC: Waist circumference
WHtR: Waist-to height ratio
ABSI: A body shape index

## References

[1] Sun H, Gong T-T, Jiang Y-T, Zhang S, Zhao Y-H, Wu Q-J (2019). Global, regional, and national prevalence and disability-adjusted life-years for infertility in 195 countries and territories, 1990–2017: results from a global burden of disease study, 2017. Aging.

[2] Hart RJ (2016). Physiological aspects of female fertility: role of the environment, modern lifestyle, and genetics. Physiol Rev.

[3] van Roode T, Dickson NP, Righarts AA, Gillett WR (2015). Cumulative incidence of infertility in a New Zealand birth cohort to age 38 by sex and the relationship with family formation. Fertil Steril.

[4] Checa Vizcaíno MA, González-Comadran M, Jacquemin B (2016). Outdoor air pollution and human infertility: a systematic review. Fertil Steril.

[5] Petraglia F, Serour GI, Chapron C (2013). The changing prevalence of infertility. Int J Gynaecol Obstet.

[6] Malina A, Suwalska-Barancewicz D (2021). Comparison of early-stage mothers and childless women seeking pregnancy: experienced stress, resilience and satisfaction with relationship with the partner. Int J Environ Res Public Health.

[7] Boivin J, Bunting L, Collins JA, Nygren KG (2007). International estimates of infertility prevalence and treatment-seeking: potential need and demand for infertility medical care. Hum Reprod.

[8] Liu J, Larsen U, Wyshak G (2005). Prevalence of primary infertility in China: in-depth analysis of infertility differentials in three minority province/autonomous regions. J Biosoc Sci.

[9] Che Y, Cleland J (2002). Infertility in Shanghai: prevalence, treatment seeking and impact. J Obstet Gynaecol.

[10] Zhou Z, Zheng D, Wu H, Li R, Xu S, Kang Y (2018). Epidemiology of infertility in China: a population-based study. BJOG.

[11] Cong J, Li P, Zheng L, Tan J (2016). Prevalence and risk factors of infertility at a rural site of Northern China. PLoS ONE.

[12] Chen J, Zhong C, Liang H, Yang Y, Zhang O, Gao E (2015). The relationship between age at menarche and infertility among Chinese rural women. Eur J Obstet Gynecol Reprod Biol.

[13] Meng Q, Ren A, Zhang L, Liu J, Li Z, Yang Y (2015). Incidence of infertility and risk factors of impaired fecundity among newly married couples in a Chinese population. Reprod Biomed Online.

[14] He Y, Zheng D, Shang W, Wang X, Zhao S, Wei Z (2020). Prevalence of oligomenorrhea among women of childbearing age in China: a large community-based study. Womens Health (Lond).

[15] Farquhar CM, Bhattacharya S, Repping S, Mastenbroek S, Kamath MS, Marjoribanks J (2019). Female subfertility. Nat Rev Dis Primers.

[16] Cohen SA, Richards CL (1994). The Cairo consensus: population, development and women. Fam Plann Perspect.

[17] Strategy to Accelerate Progress towards the Attainment of International Development Goals and Targets Related to Reproductive Health. Reprod Health Matters. 2005;13(25):11–8. 10.1016/s0968-8080(05)25166-2 16035592

[18] Inhorn MC (2009). Right to assisted reproductive technology: overcoming infertility in low-resource countries. Int J Gynecol Obstet.

[19] Bai F, Wang DY, Fan YJ, Qiu J, Wang L, Dai Y (2020). Assisted reproductive technology service availability, efficacy and safety in mainland China: 2016. Hum Reprod.

[20] Hu L, Bu Z, Huang G, Sun H, Deng C, Sun Y (2020). Assisted reproductive technology in China: results generated from data reporting system by CSRM from 2013 to 2016. Front Endocrinol (Lausanne).

[21] Vander Borght M, Wyns C (2018). Fertility and infertility: definition and epidemiology. Clin Biochem.

[22] Zegers-Hochschild F, Adamson GD, de Mouzon J, Ishihara O, Mansour R, Nygren K (2009). International Committee for Monitoring Assisted Reproductive Technology (ICMART) and the World Health Organization (WHO) revised glossary of ART terminology, 2009. Fertil Steril.

[23] Zegers-Hochschild F, Adamson GD, Dyer S, Racowsky C, de Mouzon J, Sokol R (2017). The international glossary on infertility and fertility care, 2017. Fertil Steril.

[24] The Rotterdam ESHRE/ASRM-sponsored PCOS consensus workshop group. Revised 2003 consensus on diagnostic criteria and long-term health risks related to polycystic ovary SYNDROME (PCOS). Hum Reprod. 2004;19(1):41–7. 10.1093/humrep/deh098 14688154

[25] Thoma ME, mclain AC, Louis JF, King RB, Trumble AC, Sundaram R (2013). Prevalence of infertility in the United States as estimated by the current duration approach and a traditional constructed approach. Fertil Steril.

[26] Bushnik T, Cook JL, Yuzpe AA, Tough S, Collins J (2012). Estimating the prevalence of infertility in Canada. Hum Reprod.

[27] Datta J, Palmer MJ, Tanton C, Gibson LJ, Jones KG, Macdowall W (2016). Prevalence of infertility and help seeking among 15 000 women and men. Hum Reprod.

[28] Sarac M, Koc I (2018). Prevalence and risk factors of infertility in Turkey: evidence from demographic and health surveys, 1993–2013. J Biosoc Sci.

[29] Kazemijaliseh H, Ramezani Tehrani F, Behboudi-Gandevani S, Hosseinpanah F, Khalili D, Azizi F (2015). The prevalence and causes of primary infertility in Iran: a population-based study. Glob J Health Sci.

[30] Kumar D (2007). Prevalence of female infertility and its socio-economic factors in tribal communities of Central India. Rural Remote Health.

[31] Polis CB, Cox CM, Tunçalp Ö, McLain AC, Thoma ME (2017). Estimating infertility prevalence in low-to-middle-income countries: an application of a current duration approach to Demographic and Health Survey data. Hum Reprod.

[32] Dick M-LB, Bain CJ, Purdie DM, Siskind V, Molloy D, Green AC (2003). Self-reported difficulty in conceiving as a measure of infertility. Hum Reprod.

[33] Li R, Zhang Q, Yang D, Li S, Lu S, Wu X (2013). Prevalence of polycystic ovary syndrome in women in China: a large community-based study. Hum Reprod.

[34] Zhuang J, Liu Y, Xu L, Liu X, Zhou L, Tang L (2014). Prevalence of the polycystic ovary syndrome in female residents of Chengdu. China Gynecol Obstet Invest.

[35] Hart R (2008). PCOS and infertility. Panminerva Med.

[36] Moolhuijsen LME, Visser JA (2020). Anti-Müllerian hormone and ovarian reserve: update on assessing ovarian function. J Clin Endocrinol Metab.

[37] Broer SL, Broekmans FJM, Laven JSE, Fauser BCJM (2014). Anti-Müllerian hormone: ovarian reserve testing and its potential clinical implications. Hum Reprod Update.

[38] Du X, Ding T, Zhang H, Zhang C, Ma W, Zhong Y (2016). Age-specific normal reference range for serum anti-müllerian hormone in healthy Chinese Han women: a nationwide population-based study. Reprod Sci.

[39] American College of Obstetricians and Gynecologists Committee on Gynecologic Practice and Practice Committee. Female age-related fertility decline. Committee Opinion No. 589. Fertil Steril. 2014;101(3):633–4. 10.1016/j.fertnstert.2013.12.032 24559617

[40] Testing and interpreting measures of ovarian reserve: a committee opinion. Fertil Steril. 2015;103(3):e9–17. 10.1016/j.fertnstert.2014.12.093 25585505

[41] Alborzi S, Madadi G, Samsami A, Soheil P, Azizi M, Alborzi M (2015). Decreased ovarian reserve: any new hope?. Minerva Ginecol.

[42] Henes M, Engler T, Taran F-A, Brucker S, Rall K, Janz B (2018). Ovarian cyst removal influences ovarian reserve dependent on histology, size and type of operation. Womens Health (Lond).

[43] Kasum M (2009). Fertility following myomectomy. Acta Clin Croat.

[44] Pasquali R (2006). Obesity, fat distribution and infertility. Maturitas.

[45] Loy SL, Cheung YB, Soh SE, Ng S, Tint MT, Aris IM (2018). Female adiposity and time-to-pregnancy: a multiethnic prospective cohort. Hum Reprod.

[46] Gambineri A, Laudisio D, Marocco C, Radellini S, Colao A (2019). Female infertility: which role for obesity?. Int J Obes Suppl.

[47] Kannan S, Bhaskaran RS (2019). Sustained obesity reduces litter size by decreasing proteins regulating folliculogenesis and ovulation in rats—a cafeteria diet model. Biochem Biophys Res Commun.

[48] Espinós JJ, Polo A, Sánchez-Hernández J, Bordas R, Pares P, Martínez O (2017). Weight decrease improves live birth rates in obese women undergoing IVF: a pilot study. Reprod Biomed Online.

[49] Konstantinidou F, Stuppia L, Gatta V (2020). Looking inside the world of granulosa cells: the noxious effects of cigarette smoke. Biomedicines.

[50] Lyngsø J, Kesmodel US, Bay B, Ingerslev HJ, Pisinger CH, Ramlau-Hansen CH (2021). Female cigarette smoking and successful fertility treatment: a Danish cohort study. Acta Obstet Gynecol Scand.

