# Supplementary material for: Prevalence and associated factors of infertility among 20–49 year old women in Henan Province, China
Source: Reprod Health. 2021 Dec 20;18:254. doi: 10.1186/s12978-021-01298-2 (PMC8691046; doi:10.1186/s12978-021-01298-2)

| **Supplement Table S1**. Mean and standard deviation of serum AMH grouped by age | | | | | | | |
| --- | --- | --- | --- | --- | --- | --- | --- |
| Characteristic | | Fertility(n=577) | | Infertility(n=188) | | Total(n=765) | |
|  |  | Mean | SD | Mean | SD | Mean | SD |
| Age(year) | 20-24 | 5.881 | 4.83 | 5.36 | 1.457 | 5.851 | 4.694 |
|  | 25-29 | 4.682 | 3.557 | 6.169 | 4.965 | 4.864 | 3.772 |
|  | 30-34 | 4.549 | 4.153 | 4.512 | 3.803 | 4.538 | 4.049 |
|  | 35-39 | 2.541 | 1.741 | 2.497 | 2.188 | 2.525 | 1.908 |
|  | 40-44 | 1.524 | 1.385 | 1.728 | 2.127 | 1.576 | 1.595 |
|  | 45-49 | 0.615 | 0.971 | 0.692 | 1.076 | 0.635 | 0.986 |

**Supplement Figure S1**. (A) The changes of infertility rate with age and age of marriage. The infertility rate = The number infertile / The total number by age group (no participants got married at 35-39 years or 45-49 years old). (B) The different rate of women who had protected sexual intercourse and who still hoped to conceive in the future by age group.


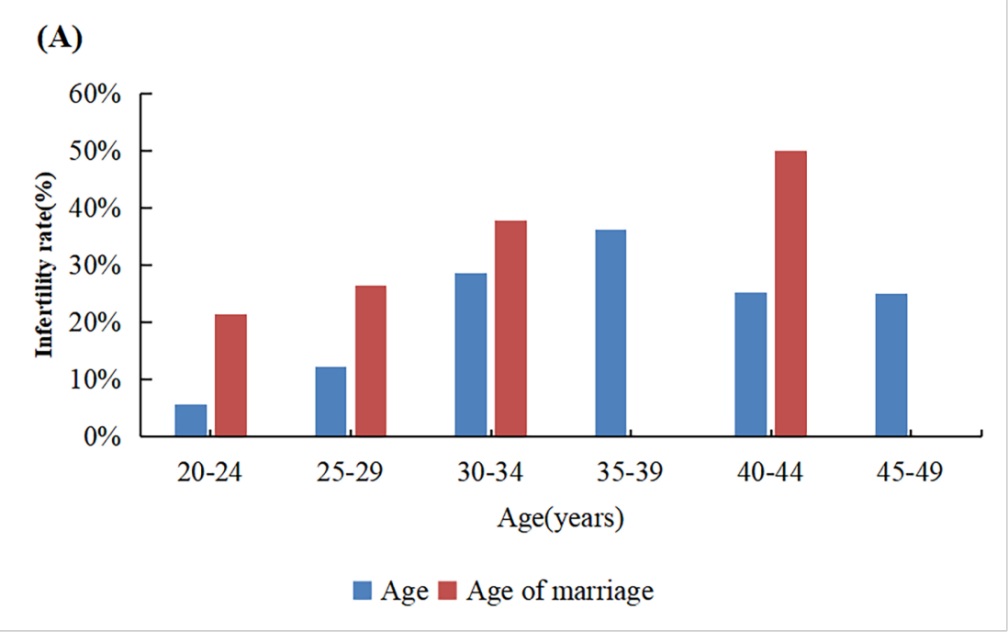

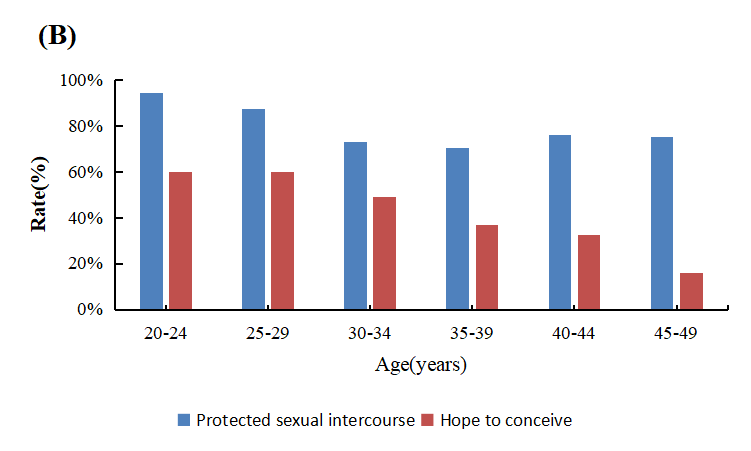


**Supplement Figure S2.** (A) The percentage of infertile women went to hospital seeking medical help. (B) The main cause of infertility according to the women who sought medical help.


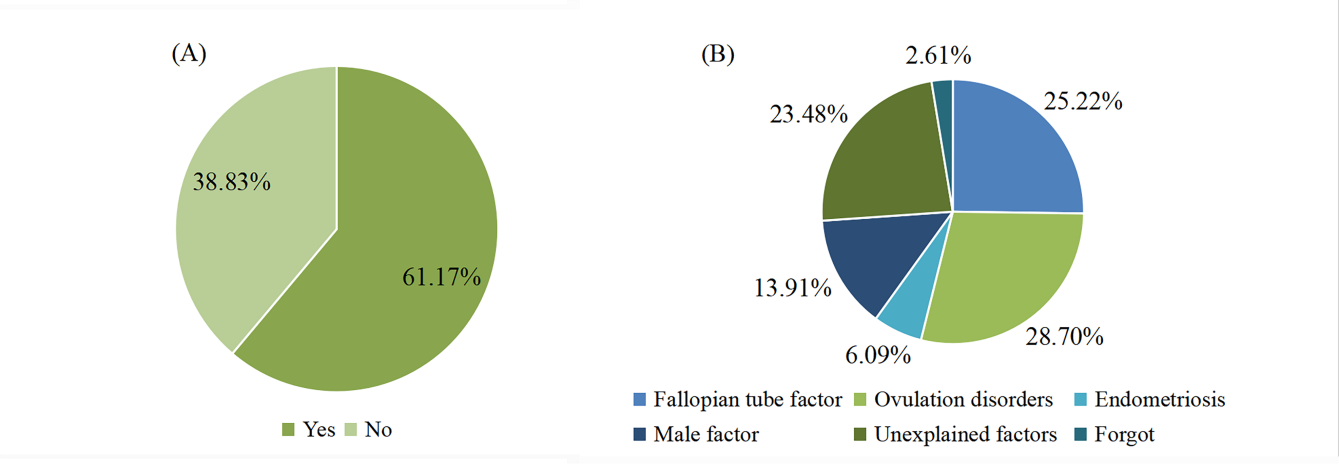

Supplement: Supplementary file 1 — Additional file 1. Supplement Table S1. Mean and standard deviation of serum AMH grouped by age. Supplement Figure S1. (A) The changes of infertility rate with age and age of marriage. The infertility rate = The number infertile / The total number by age group (no participants got married at 35-39 years or 45-49 years old). (B) The different rate of women who had protected sexual intercourse and who still hoped to conceive in the future by age group. Supplement Figure S2. (A) The percentage of infertile women went to hospital seeking medical help. (B) The main cause of infertility according to the women who sought medical help. [file 12978_2021_1298_MOESM1_ESM.docx]
